# Further Research in Food Processing Safety and Quality from Field to Fork

**DOI:** 10.3390/foods14040546

**Published:** 2025-02-07

**Authors:** Tamara Lazarević-Pašti, Nebojša Potkonjak

**Affiliations:** VINČA Institute of Nuclear Sciences—National Institute of the Republic of Serbia, University of Belgrade, Mike Petrovica Alasa 12–14, 11000 Belgrade, Serbia; npotkonjak@vin.bg.ac.rs

This Editorial refers to the Special Issue “Further Research in Food Processing Safety and Quality from Field to Fork”, which addresses critical aspects of ensuring the safety and quality of food throughout the entire production chain. Modern food processing technologies enhance product quality and contribute to reducing waste, maximizing the use of byproducts, and promoting sustainable practices. Advances in green extraction, pesticide residue assessment, and novel sensor technologies are crucial for undertaking contemporary food safety challenges. These approaches align with global efforts to reduce environmental impact, ensure regulatory compliance, and improve consumer trust in food systems. This Special Issue highlights the importance of innovative technologies and strategies for improving food safety and minimizing risks to public health from agricultural practices to final processing stages.

The presented collection of research focuses on investigations aimed at the following:

(i) Advanced methodologies for detecting and mitigating contaminants, such as pesticides, mycotoxins, and heavy metals, in food products;

(ii) Evaluate the impact of pretreatment, processing, and storage conditions on food quality and safety attributes;

(iii) Promote the valorization of agro-industrial byproducts through green and sustainable extraction techniques;

(iv) Explore innovative materials and sensors for real-time monitoring and quality assurance in food systems.

This collection includes 15 thoroughly reviewed manuscripts, each offering valuable insights into diverse aspects of food processing safety and quality. The contributions collectively underline the necessity of integrating advanced technologies, interdisciplinary approaches, and sustainability principles to ensure safe and high-quality food from field to fork.

Al-Hamry et al. [1] developed multi-modal sensors based on PDAC/rGO films for detecting dimethoate, demonstrating their real-time efficiency in food safety monitoring. In the field of pesticide removal, Tasić et al. [2] investigated viscose-derived activated carbon fibers for the adsorption of malathion and chlorpyrifos from liquid food samples, highlighting their high efficiency, selectivity, and reusability. Similarly, Milanković et al. [3] explored using spent coffee grounds as a sustainable adsorbent for organophosphate pesticide removal, showing promising adsorption capacities and safe application in food and water treatment.

Aside from detection and removal, regulatory aspects and degradation strategies are also examined. Leskovac and Petrović [4] reviewed pesticide contamination concerns, focusing on organophosphates, addressing regulation gaps, and highlighting the need for global harmonization of food safety standards. Flamminii et al. [5] studied the impact of washing, blanching, freezing, and frozen storage on pesticide residues in spinach, identifying optimal processing conditions to minimize contamination. Lastly, Jocić et al. [6] proposed a green pretreatment strategy using ionic liquid-based aqueous two-phase systems for pesticide extraction from strawberries, achieving high extraction efficiencies while maintaining sustainability.

While pesticide-related food safety remains critical, other contaminants such as toxic elements, pharmaceutical residues, and pathogens also pose significant health risks. Several studies have explored these concerns, focusing on their occurrence, impact, and potential mitigation strategies. Paucar-Quishpe et al. [7] assessed human exposure to ivermectin residues in bovine-derived foods, revealing that while contamination levels in meat were negligible, milk samples exceeded safety limits, highlighting the need for stricter regulations and monitoring. Cantoral et al. [8] investigated dietary cadmium exposure in Mexico, identifying high contamination levels in certain vegetables and cocoa products, with children exceeding the tolerable weekly intake, emphasizing the necessity of food safety policies and surveillance programs.

Besides toxic element contamination, microbial hazards and their health effects are also explored. Mafe and Büsselberg [9] reviewed the carcinogenic risks of mycotoxins in food, detailing their sources, health implications, and current detection and control strategies while also calling for technological advancements to enhance food safety. Similarly, Mafe and Büsselberg [10] examined foodborne pathogens and their metabolites, particularly their role in cancer development, highlighting detection challenges and the importance of innovative control strategies.

Finally, Savić et al. [11] developed an advanced analytical method for determining rare earth elements in coffee, demonstrating their presence in varying concentrations but concluding that they pose a negligible health risk to consumers.

Beyond contamination concerns, food processing and storage methods play a crucial role in determining food products’ safety, quality, and nutritional value. Lučić et al. [12] examined how ultrasound and chemical pretreatments affect the quality of dried red pepper, revealing that ultrasonic treatment altered morphology. At the same time, potassium metabisulfite was more effective than citric acid in preserving color, antioxidant activity, and overall quality. Augustyńska-Prejsnar et al. [13] studied the impact of temperature and storage duration on post-laying hen meat, finding that lower temperatures (2 °C) better maintained microbiological and sensory quality, prolonging shelf life compared to storage at 6 °C.

Innovative extraction methods for bioactive compounds are also explored. Dimitrijević et al. [14] developed a green extraction method for polyphenols from grape byproducts using aqueous biphasic systems, achieving high extraction efficiency (~99% for resveratrol and 78% for gallic acid), demonstrating the potential for sustainable polyphenol recovery.

Pęksa et al. [15] investigated glycoalkaloid content in potato snacks made from colored potatoes, assessing the effects of organic acid soaking and thermal processing. Malic acid and the purple-fleshed variety were most effective in reducing α-chaconine levels, with all processing methods significantly lowering toxic glycoalkaloid concentrations.

In conclusion, the studies presented in this Special Issue provide substantial contributions to the field of food safety, quality, and sustainability. Each study emphasizes innovative methods for assessing food contaminants, such as pesticides, mycotoxins, and heavy metals, as well as the impact of foodborne pathogens and their metabolites. From developing efficient extraction techniques, including ultrasound-assisted extraction and ionic liquid-based systems, to evaluating potential health risks associated with food contaminants, the research highlights the importance of optimizing analytical methods and ensuring consumer safety. Furthermore, these studies emphasize the role of advanced technologies in improving food monitoring systems and enhancing the overall sustainability of food production. Future research should focus on refining these methods, particularly in sensor development, analytical precision, and risk assessment, to further enhance food safety, regulatory compliance, and environmental sustainability.

## Data Availability

Not applicable.
